# Effects of refundable state earned income tax credits on access to medical and dental services of low-income mothers

**DOI:** 10.1016/j.ssmph.2025.101787

**Published:** 2025-03-19

**Authors:** Haobing Qian, George L. Wehby

**Affiliations:** a Department of Family and Community Medicine, University of California, San Francisco, CA, United States; b Department of Health Management and Policy, College of Public Health, University of Iowa, Iowa City, IA, United States; c Department of Economics, University of Iowa, Iowa City, IA, United States; d Preventive & Community Dentistry, University of Iowa, Iowa City, IA, United States; e Public Policy Center, University of Iowa, Iowa City, IA, United States; f National Bureau of Economic Research, Cambridge, MA, United States

**Keywords:** Health equity, Social welfare policies, Maternal health, Access to medical and preventive care

## Abstract

**Background:**

Low-income women face constraints to timely access to medical and dental services. There is little evidence on whether refundable state earned income tax credit (EITC) programs affect access to care. We examine the effects of refundable state EITC levels on accessing medical and dental care among low-income mothers and potential interactions with state Medicaid eligibility levels.

**Methods:**

We use data from 1996-2019 Behavioral Risk Factor Surveillance System (BRFSS). We focus on single mothers aged 18–44 with high school or less education and two or more children as the group that receives the most EITC payments, but also consider other subgroups. We consider the timing of EITC disbursement relative to interview month and outcome measurement. The regression analysis adjusts for state time-invariant differences, national time trends, and several individual-level and state time-varying covariates.

**Results:**

There is little evidence that higher refundable state EITC affects access to medical and dental services among low-income mothers including among the group mostly likely to benefit from this policy. A small improvement in dental visits and decrease in forgone medical visits are observed in some models. However, these results are sensitive to the timing of EITC measure and included interview months. Moreover, some effects are observed among subgroups less exposed to EITC. There is also no evidence that EITC effects differ by state Medicaid eligibility.

**Conclusion:**

The overall small payments from refundable state EITC do not appear to impact medical and dental care access of low-income mothers. Further research to understand potential individual-level heterogeneity by EITC amounts and timing relative to health care needs is important.

## Highlights

• Low-income women of childbearing age face constraints in accessing medical and dental care.
• We examine effects of refundable EITC on access to care among low-income mothers irrespective of pregnancy.
• There is little evidence that the small refundable state EITC affects access including the subgroup receive most benefits.
• There is also no evidence that the EITC effects on access vary with the state Medicaid eligibility level.
• These findings add evidence on EITC effects on access which largely focuses on federal or state EITC among pregnant women.

## 1 Introduction

Low-income non-elderly adults including women of childbearing age in the United States have less access to medical and dental services than those at higher income. In 2014, nearly 33 % of those below 100 % of the federal poverty level (FPL) did not receive needed health care due to cost, compared to about 20 % between 100 and 400 % FPL, and 5 % above 400 % FPL (Okoro et al., 2017). Similarly in 2010, 1 in 4 of those below 200 % FPL had never had a dental visit or returned for one for over 5 years, compared to less than 1 in 10 at higher income (Licata & Paradise, 2012, pp. 1–8). Moreover, while access to medical care and dental services of low-income non-elderly adults including women of childbearing age generally improved in states that expanded Medicaid eligibility under the Affordable Care Act (Lyu et al., 2020; Lyu & Wehby, 2019, 2021; Margerison et al., 2020; Wehby et al., 2019), income disparities in access remain substantial (Griffith et al., 2017).

Consistent and continuous access to comprehensive insurance coverage of preventive services may improve the health, especially for Medicaid-eligible and low-income people who may need more support (Semprini et al., 2020). Conceptually, greater income may help in covering out-of-pocket health care expenses, whether direct costs (including cost sharing in private coverage) or related costs such as transportation or childcare expenses. Out-of-pocket costs represent a large barrier to care (Riggs & Ubel, 2014), and more so for dental services than medical services due to less access to dental insurance coverage through either private or public plans.

The Earned Income Tax Credit (EITC) is one of the largest antipoverty programs in the United States that provides a cash transfer to low-income working families through a fixed annual lump-sum tax refund. The majority of federal EITC is disbursed during February and March (LaLumia, 2013) and is spent down quickly within three months of receipt (Souleles, 1999). Over half of the states have also enacted their own EITC programs that follow similar eligibility rules with smaller credits as a percentage of the federal credit (Center on Budget and Policy Priorities, 2021). Most states provide refundable EITC programs with substantial variation (Center on Budget and Policy Priorities, 2023).

Single mothers represent the largest share of credit recipients (Eissa & Hoynes, 2011; Hoynes & Patel, 2018). Studies have shown that EITC from federal and state programs improve maternal health (Averett & Wang, 2013; Boyd-Swan et al., 2016; Cowan & Tefft, 2012; Evans & Garthwaite, 2014; Gangopadhyaya et al., 2020; Hamad & Rehkopf, 2015; Hoynes et al., 2015; Markowitz et al., 2017; Qian & Wehby, 2021; Rehkopf et al., 2014). There is however less evidence on effects of state EITC programs on health care access and utilization. One study found state EITC to be associated with more prenatal care visits (Strully et al., 2010), although another study using more recent data found little evidence for that effect (Markowitz et al., 2017). One study combining federal and state EITC did not find any short-term effects on out-of-pocket spending on health care among EITC eligible adults (Hamad & Niedzwiecki, 2019). Also, little is known about whether the effects of EITC on health care access and utilization differ by Medicaid eligibility. Understanding whether these programs have synergetic effects is important since they both target low-income adults and the benefits they offer may have complementary effects on health care access and utilization.

This study examines the effects of refundable state EITC programs on access to medical and dental services among low-income mothers. We consider potential differences in the timing of these effects relative to the disbursement period and when outcomes are measured. We also evaluate whether EITC effects interact with those of Medicaid eligibility. We evaluate refundable credits because previous work shows refundable rather than non-refundable credits are related to improvement in maternal heath (Markowitz et al., 2017, Qian and Wehby, 2021, Qian and Wehby, 2023). Non-refundable programs are less beneficial for very low-income individuals if their tax liability is less than the credit (Johnson, 2001). And as noted above, most state EITC programs are refundable. We focus on single mothers of childbearing age who have two or more children because they receive the most credit on average but consider other subgroups in additional analyses.

We employ nationally representative data over nearly two decades. Over the study period, the number of states with refundable EITC increased from 4 to 22 programs, and average refundable credits increased from 15 % to 25 % of the federal credit, with a range from 3.5 % to 85 %, indicating substantial within and between state variation to evaluate effects of this policy on access.

## 2 Methods

### 2.1 Data and sample

Data comes from the Behavioral Risk Factor Surveillance System (BRFSS) from 1996 (the first year of annual data on state Medicaid eligibility for parents that we can readily access, which is a main variable we interact with EITC refundable credits as described below) through 2019. BRFSS is a repeated cross-sectional survey of a nationally representative sample (Behavioral Risk Factor Surveillance System, 2024). It collects data on individual-level demographic, health, and behaviors.

We focus in the main sample on single mothers aged 18–44 years with a high school degree or less who have at least two children following prior literature since they have the highest proportion of receiving EITC benefits and the largest EITC return on average (Evans & Garthwaite, 2014; Hoynes et al., 2015; Markowitz et al., 2017; Qian & Wehby, 2021; Strully et al., 2010). To avoid sample selection bias, we select the sample based on education rather than income and employment because these variables are potentially endogenous to EITC.

The study design and analysis described below estimate an intent-to-treat effect of EITC, i.e. the average effect including both EITC recipients and non-recipients irrespective of actual credit received. Therefore, it is important to focus on the group most likely to be affected by EITC to estimate this effect. Because EITC eligibility is based on family income and number of children (higher eligibility and payments for lower income and 2 or more children), other groups such as single mothers with one child or married mothers or those with higher education or fathers with children have lower proportions who receive maximum EITC benefits and smaller average payments. Family income is higher on average among married women and those with higher education, so both the proportion eligible and the credit amount are less. For example, 88 % of single mothers aged 18–44 years with high school or lower education who have two or more children in BRFSS in years 1992–2017 in states with refundable EITC were reported to have been eligible for EITC and have average payment of $520 including both recipients and non-recipients. In contrast, 78 % of single mothers with the same education and age and one child in the same data were reported to have been eligible for EITC with an average payment of $344 (Qian & Wehby, 2021). If the same EITC payment has similar effects across subgroups, then the intent-to-treat estimates of EITC effects are expected to be smaller in the subgroups with lower proportions of EITC eligible individuals and smaller average payments. Therefore, effects are expected to be small in groups that have low eligibility rates and payments such as high-educated married mothers whose family incomes make them ineligible for credits or for small credit amounts; any effects in such groups would likely be spurious. It is also important to reduce the number of tests to avoid multiple testing bias. Nonetheless, we consider other subgroups of mothers in additional analyses based on marital status (single or married), number of children (1 or 2+), and educational level (high school or less versus above high school). We also evaluate EITC effects for single childless women with high school or less education who receive little state EITC benefit ($33 on average) (Qian & Wehby, 2021) in a placebo check.

### 2.2 Study measures

#### 2.2.1 EITC measure

The EITC measure is the refundable state EITC level as a percentage of the federal credit, obtained from the National Bureau of Economic Research for tax years before 2000 (National Bureau of Economic Research, 2019) and Urban-Brookings Tax Policy Center for tax year 2000 through 2018 (Tax Policy Center, 2020). This measure captures EITC generosity in states with refundable programs (Gangopadhyaya et al., 2020; Markowitz et al., 2017). States select their EITC level as a fixed percent of the federal EITC independently of the individual's eligibility criteria. In that way, this measure also reflects differences in the maximum credit between states. We assign 0 on this measure to states without EITC and states with non-refundable EITC. We also assign 0 to Washington because it did not implement its EITC program and exclude Maryland because it offers a mixed program. Supplemental Table S1 shows the number of states with refundable EITC programs and average credit levels over tax years 1995–2018 (corresponding to BRFSS interview years 1996–2019).

#### 2.2.2 Outcomes

We study three access outcomes. *Forgone medical visit* is a binary (0/1) indicator for whether the person needed but did not see a doctor within the past 12 months due to cost. *Routine medical checkup* is a 0/1 binary indicator for whether the person had a routine medical checkup within the past 12 months. Both of these outcomes are asked each survey year. *Dental visit* is a binary (0/1) indicator for whether the person had a dental visit within the past 12 months. This question is only included in even years of the survey from 2002 to 2018 and is not included in odd years (CDC - BRFSS Annual Survey Data, 2024).

#### 2.2.3 Covariates

Individual-level covariates include a 0/1 indicator for five age categories (18–24, 25–29, 30–34, 35–39, or 40–44 years), education level (high school or less), race/ethnicity (non-Hispanic White, non-Hispanic Black, Hispanic, or other race/ethnicity), and a 0/1 indicator for 3 or more children versus 2. State-level (time-varying) covariates include the maximum state Temporary Assistance for Needy Family (TANF) monthly payment for a family of 3 (University of Kentucky Center for Poverty Research, 2025), real minimum wage averaged over the past 12 month period (United States Department of Labor Wage and Hour Division, 2020), Medicaid income eligibility for parents as percent of the FPL (Hamersma & Kim, 2009), median household income, and unemployment rate (United States CensusBureau, 2019).

### 2.3 Statistical analysis

We estimate the effects of refundable state EITC programs using a two-way fixed effects regression which is a form of a Difference-in-Differences model. The model utilizes within-state variation in EITC levels over time and flexibly accommodates differences in the timing of EITC enactment and changes in generosity across states. It also controls for time-invariant unobserved confounders between states, and national time trends in outcomes shared across states. The model compares states with changes in refundable EITC (including enacting a new program or modifying credit levels) to other states without such changes over the same period to estimate effects of refundable EITC levels. The model is specified as follows:

(1) Y_ist_ = β_0+_ β_1_REFUND_EITC_st_ + β_2_X_ist_ +β_3_P_st_ + θ_s_ + λ_t_ + e_ist_

In model (1), Y_ist_ is one of the three access outcomes for mother i in state s in survey year t. REFUND_ EITC_s_ is the refundable state EITC as a percent of the federal EITC (scaled in 10 percent points). X_ist_ are individual-level sociodemographic variables described above, plus fixed effects (0/1 indicators) for month of survey. P_st_ are state-specific time-varying covariates described above. θ_s_ includes state fixed effects (capturing state-level time-invariant confounders) and λ_t_ includes survey year fixed effects (capturing shared time trends between states).

**Fig. 1:**
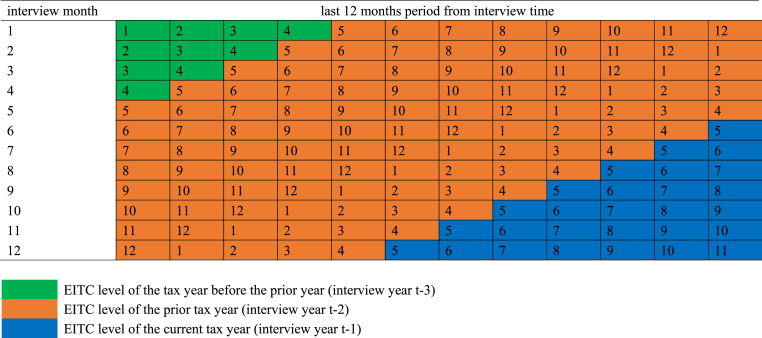
EITC assignment depending on the interview months following the disbursement.

Because the outcomes are reported over the past 12 months from the interview month, and because most EITC payments are disbursed within the first quarter (specifically February, March, and April) of the year based on the EITC level of the prior tax year (LaLumia, 2013; Souleles, 1999), it is important to consider the timing of EITC disbursement relative to the interview month and to check the sensitivity of results to different approaches to capture this variation in timing. Therefore, we consider three approaches to measure REFUND_EITC. The first approach calculates an average of the EITC levels over 12 months before the interview month. We assign to each of those 12 months a tax year and its corresponding EITC level depending on which disbursement period the month follows as illustrated in Fig. 1. For May through December, the EITC level of the prior tax year is assigned. For January through April, the EITC level of the tax year before the prior year is assigned. In a given interview year, the average EITC level varies depending on the interview month because of the difference in the past 12 months. For example, for an interview in January of 2000, the past 12 months are January through December of 1999. Of those 12 months, 4 months (January–April) are each assigned the EITC level from tax year 1997 since disbursement from tax year 1998 which would occur in those four months might not yet have been received and spent to influence outcomes in those months. For the 8 months from May through December of 1999 in this example, the EITC level from tax year 1998 is assigned since these months follow the disbursement period for that tax year (which again is the first quarter of the year).

One limitation of this first approach to measuring EITC levels is assuming that EITC recipients smooth the spending of payments across the past 12 months which is unlikely; some evidence suggests that payments are spent within 3 months from receipt ((LaLumia, 2013; Souleles, 1999). Another limitation is assuming that respondents recall events similarly across each of the past 12 months and any recall errors are independent of the event timing in this 12-month window relative to the interview month. This assumption may also not hold considering recall errors in self-reported utilization of health services and that respondents are more likely to report more recent events of utilization (Bhandari & Wagner, 2006; Jobe, 2003; Ritter et al., 2001; Schmitz et al., 2002; Seidl et al., 2016; Wolinsky et al., 2014).

**Fig. 2:**
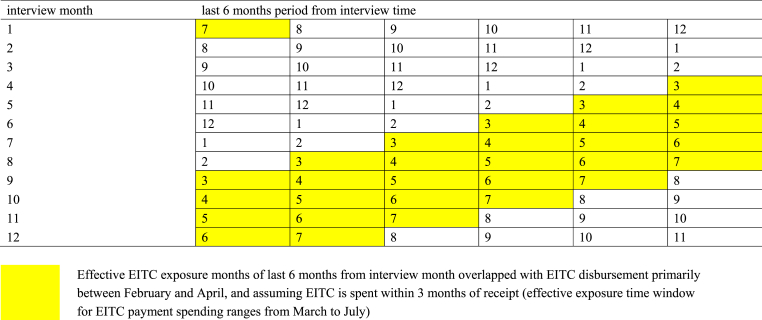
Effective EITC exposure time window for different interview months.

To address these issues in an alternative approach to measuring EITC levels, we focus on individuals interviewed after the expected disbursement period (first quarter) and assign the EITC level from the prior tax year (i.e. the year before the interview year). We consider two scenarios in this approach. One scenario includes interviews from May through December. The other scenario includes interviews from June through November. Even though outcomes are still reported over the past 12 months for respondents interviewed later in the year in these scenarios, more of the recent months before the interview month occur following the latest disbursement period than when including respondents from earlier months in the year. If outcomes primarily reflect the experiences of more recent months and EITC payments are spent within a short period after disbursement, focusing on individuals interviewed after the first quarter might more accurately capture exposure to EITC. Considering that EITC is typically disbursed between February and April, and assuming that EITC payments are spent within 3 months from receipt, the exposure window for EITC payment spending would range from March through July. Assuming that the past 6 months from the interview month are most relevant for reporting the outcomes, there would be 2 to 5 of these 6 months (depending on interview month) that would overlap with the EITC spending window for interviews between May and December (Fig. 2). Similarly, there would be 3–5 months of this overlap for interviews between June and November and therefore more EITC exposure. In brief, we would expect that there is more exposure to EITC payments and their effects on outcomes in those two scenarios that limit the sample to interviews after the last disbursement period than the first approach including interviews from all months.

Lastly, to examine the interaction between refundable EITC and state Medicaid income eligibility for parents, we re-estimate the above model adding a binary indicator for above versus below state-level median eligibility over the study period in the full analytical sample (81 % based on the weighted data) and its interaction with the EITC measure:

(2) Y_ist_ = c_0_ + c_1_REFUND_EITC_st_ + c_2_MEDICAID_st+_c_3_REFUND_EITC_st_ × MEDICAID_st_ + c_4_X_ist_ + c_5_Z_st_ + θ_s_ + λ_t_ + u_ist._

In model (2), MEDICAID indicates that the state has parental income eligibility above 81 % of the FPL (0 versus below). Z includes the state controls defined above except for parental income eligibility for Medicaid (replaced by the binary indicator). c_3_ estimates the interaction between refundable state EITC and above-median Medicaid eligibility.

All models are estimated using weighted least squares with BRFSS sampling weights and standard errors are clustered at the state level. We consider estimates to be statistically significant at p < 0.05.

## 3 Results

### 3.1 Descriptive analysis

The number of observations in the primary analytical sample of mothers with high school or less education and at least two children who had complete data on the outcomes and all covariates varied between 50445 and 99747 depending on the outcome. Each year, that sample included between 2500 and 5000 observations except 2001 when it had about 500 observations. In the primary sample, 28.3 % reported forgoing needed medical care due to cost, 66 % reported having a routine medical checkup, and about 58.7 % reported having a dental visit, all measured over the past 12 months. Nearly 40 % had less than high school education and 54 % had two children (Table 1).

**Table 1:** Descriptive statistics of medical and dental access and demographic characteristics of the analytical sample, single low-educated women 18–44 with two or more children in BRFSS.

	Mean or Frequency (%)
**Access outcomes (during past 12 months)**	
Foregone medical visit	28.3
Routine medical checkup	66.0
Dental visit	58.7

**Demographic control variables**	
Age in 5 years categories	
18-24	32.1
25-29	18.7
30-34	21.9
35-39	16.6
40-44	10.7
Race/ethnicity	
Black, non-Hispanic	22.0
White, non-Hispanic	35.4
Hispanic	37.6
Other race/ethnicity	5.0
Education	
Less than High school	40.3
High school graduates	59.7
Number of children	
2 children	53.7
3 or more children	46.3

Note: BRFSS sampling weights are applied. The sample size for each outcome varies depending on missing data. The descriptive statistics for the control variables and for forgone care due to cost are based on the sample with complete data on all those variables (N = 99747). The sample size for any routine checkup during last 12 months is based on sample with complete data on that outcome and control variables (N = 88967). The sample size for dental visits during last 12 months is based on sample with complete data on that outcome and control variables (N = 50445). Descriptive statistics are based on annual data from years 1996–2019 except for the any dental visits outcome, which is measured biannually in even years from 2002 through 2018.

### 3.2 Main estimates of refundable EITC effects

Table 2 reports the regression estimates of refundable EITC effects separately for the three EITC measures and samples described above. When averaging EITC levels over the past 12 months and including respondents from all interview months, refundable EITC is associated with small but statistically significant increase in *forgone medical visit* and decline in *routine medical checkup.* Specifically, a 10 percentage-point increase in refundable state EITC is associated with 0.3 percentage-point increase in likelihood of *forgone medical visit* and 0.6 percentage-point reduction in likelihood of *routine medical checkup*. These estimates might be considered counterintuitive as they suggest that the higher income from EITC is associated with reduced access and utilization of medical care. In contrast, this measure of EITC is not associated with *dental visit*.

**Table 2:** Effects of a ten-percentage-point increase in refundable state EITC (as % of federal credit) on access to medical and dental care, single low-educated women aged 18–44 Years with two or more children in BRFSS.

Model Specifications	(1) Averaging EITC across the past 12 months and including all interview months	(2) Assigning EITC based on prior tax year level and including May–December interviews	(3) Assigning EITC based on prior tax year level and including and including June–November interviews	Outcome Mean
	Effect (SE)	Effect (SE)	Effect (SE)	
Foregone medical visit	0.003∗	−0.002	−0.006∗	0.3
	(0.002)	(0.003)	(0.003)	
Routine medical checkup	−0.006∗∗	−0.003	−0.002	0.7
	(0.002)	(0.002)	(0.001)	
Dental visit	0.0005	0.01∗	0.01	0.6
	(0.003)	(0.004)	(0.005)	

Note: Each cell represents the effect of a 10 percentage-point increase in refundable state EITCs (relative to federal credits) on the likelihood of an outcome on a 0–1 probability scale; multiplying this coefficient by 100 gives the likelihood change in percentage points. Estimates for forgone care and any routine checkups are based on annual data from BRFSS 1996–2019. Estimate for any dental visits is based on biannual data in even years from 2002 through 2018. Specification (1) includes one EITC variable, average of the refundable percentage of federal credit over 12 months before the interview month depending on which disbursement period the month follows; specification (2) and (3) includes one EITC variable, the refundable percentage of federal credit from the prior tax year (states with no EITC and states with non-refundable ETIC have 0 on this variable as the control group). All models control for state, year, and month of survey fixed effects. The demographic controls include indicators for maternal age, an indicator for mothers of three or more children versus mothers of two children only, race/ethnicity, and education. State level controls include average state minimum wage from the past 12 months, state unemployment rate, state median household family income, maximum state TANF monthly payment for a family of 3, and Medicaid parental eligibility as % FPL. The regression is estimated using weighted least squares using BRFSS sampling weights; standard errors (SE) are clustered at state level and shown in parentheses. ∗*p* < 0.05, ∗∗*p* < 0.01.

When limiting the sample to interviews from May through December and assigning the EITC level of the prior tax year, the EITC effect estimates are smaller and no longer statistically significant for *forgone medical visit* and *routine medical checkup*. There is also a statistically increase in likelihood of *dental visit* by 1 percentage-point with each 10-percentage-points of EITC.

Lastly, when limiting the sample to interviews from June through November and assigning the prior tax year EITC, there is a statistically significant decline in likelihood of *forgone medical visit* by 0.6 percentage-points per 10 EITC percentage-points and no association with *routine medical checkup*. The estimate for *dental visit* is similar to that from the May–December interview sample but is not statistically significant.

### 3.3 Estimates of interactive effects of refundable EITC and medicaid

Table 3 shows estimates of the interactions between refundable state EITC levels and an indicator for a state's Medicaid income eligibility for parents exceeding the median eligibility level across states. These estimates are obtained separately for each of the three EITC measures and samples described above. For all three EITC measures, there are no statistically significant interactions with above-median state Medicaid eligibility. Noteworthy however that for all outcomes and EITC measures, the signs of the interactions are suggestive of complementary (reinforcing) rather than substitution (offsetting) effects, albeit again not statistically significant.

**Table 3:** Effects of a ten-percentage-point increase in refundable state EITC (as % of federal credit), medicaid parental eligibility, and their interaction on access to medical and dental care, single low-educated women aged 18–44 Years with two or more children in BRFSS.

Model Specifications	(1) Averaging EITC across the past 12 months and including all interview months			(2) Assigning EITC based on prior tax year level and including May–December interviews			(3) Assigning EITC based on prior tax year level and including and including June–November interviews			Outcome mean
	10 percentage-point increase in refundable EITC	Above median state Medicaid parent eligibility (>81 % of FPL)	Interaction between refundable EITC increase and above median Medicaid eligibility	10 percentage-point increase in refundable EITC	Above median state Medicaid parent eligibility (>81 % of FPL)	Interaction between refundable EITC increase and above median Medicaid eligibility	10 percentage-point increase in refundable EITC	Above median state Medicaid parent eligibility (>81 % of FPL)	Interaction between refundable EITC increase and above median Medicaid eligibility	
	Effect (SE)	Effect (SE)	Effect (SE)	Effect (SE)	Effect (SE)	Effect (SE)	Effect (SE)	Effect (SE)	Effect (SE)	
Foregone medical visit	0.008 (0.01)	−0.02 (0.01)	−0.006 (0.01)	0.006 (0.01)	−0.003 (0.02)	−0.009 (0.01)	−0.0009	−0.004	−0.005	0.3
							(0.01)	(0.02)	(0.01)	
Routine medical checkup	−0.02 (0.01)	0.02 (0.01)	0.01 (0.01)	−0.01 (0.01)	0.001 (0.01)	0.010 (0.01)	−0.01	−0.007	0.010	0.7
							(0.01)	(0.02)	(0.01)	
Dental visit	−0.02 (0.02)	−0.009 (0.01)	0.02 (0.02)	−0.004 (0.02)	−0.01 (0.01)	0.01 (0.02)	−0.01	−0.04	0.02	0.6
							(0.02)	(0.02)	(0.02)	

Note: Each row represents estimates from one regression on the likelihood of an outcome on a 0–1 probability scale; multiplying this coefficient by 100 gives the likelihood change in percentage points. Estimates for forgone care and any routine checkups are based on annual data from BRFSS 1996–2019. Estimate for any dental visits is based on biannual data in even years from 2002 through 2018. Specification (1) includes one EITC variable, average of the refundable percentage of federal credit over 12 months before the interview month depending on which disbursement period the month follows; specification (2) and (3) includes one EITC variable, the refundable percentage of federal credit from the prior tax year (states with no EITC and states with non-refundable ETIC have 0 on this variable as the control group), a 0/1 indicator for state parental Medicaid eligibility above 81 % of the FPL (weighted median in the sample), and their interaction. All models control for state, year, and month of survey fixed effects, and for demographic and state time-varying variables. The demographic controls include indicators for maternal age, an indicator for mothers of three or more children versus mothers of two children only, race/ethnicity, and education. State level controls include average state minimum wage from the past 12 months, state unemployment rate, state median household family income, and maximum state TANF monthly payment for a family of 3. The regression is estimated using weighted least squares using BRFSS sampling weights; standard errors (SE) are clustered at state level and shown in parentheses. ∗*p* < 0.05, ∗∗*p* < 0.01.

### 3.4 Alternate groups and placebo checks

Table 4 reports the EITC effect estimates for other subgroups of mothers by marital status, education level, and number of children. Specifically, we add the following subgroups of single mothers: high school or less education with 1 child, above high school education with 1 child, and above high school education with 2 or more children. We also add similar subgroups for married mothers in addition to a subgroup of high school or less education and 2 or more children.

**Table 4:** Effects of a ten-percentage-point increase in refundable state EITC (as % of federal credit) on access to medical and dental care, different subgroups of mothers in BRFSS.

Model specifications	(1) Averaging EITC across the past 12 months and including all interview months	(2) Assigning EITC based on prior tax year level and including May–December interviews	(3) Assigning EITC based on prior tax year level and including and including June–November interviews	Outcome Mean
	Effect (SE)	Effect (SE)	Effect (SE)	
**Single Mothers**				

*Single Low Educated Mothers with 1 Child*				

Foregone medical visit	−0.003∗	0.0005	−0.004	0.3
	(-0.001)	(-0.002)	(0.003)	
Routine medical checkup	0.003	−0.002	0.003	0.7
	(-0.003)	(0.003)	(0.003)	
Dental visit	0.003	−0.003	0.002	0.6
	(-0.003)	(0.005)	(0.006)	
*Single above High School Educated Mothers with 2 or More Children*				

Foregone medical visit	−0.001	−0.002	−0.002	0.2
	(-0.002)	(0.003)	(0.003)	
Routine medical checkup	0.002	−0.001	−0.002	0.7
	(-0.002)	(0.002)	(0.002)	
Dental visit	−0.01∗∗∗	−0.009∗∗	−0.006	0.7
	(-0.003)	(0.003)	(0.003)	
*Single above High School Educated Mothers with 1 Child*				

Foregone medical visit	−0.002	0.0007	0.002	0.2
	(-0.002)	(0.002)	(0.003)	
Routine medical checkup	−0.007∗∗	−0.01∗∗∗	−0.01∗∗∗	0.7
	(-0.002)	(0.002)	(0.003)	
Dental visit	0.004	0.0006	−0.002	0.7
	(-0.003)	(0.003)	(0.003)	
**Married Mothers**				

*Married Low-Educated Mothers with 2 or More Children*				

Foregone medical visit	−0.002	−0.0005	0.0004	0.2
	(-0.002)	(0.002)	(0.001)	
Routine medical checkup	−0.009∗	−0.01∗∗	−0.01∗∗	0.6
	(-0.004)	(0.004)	(0.005)	
Dental visit	−0.007∗	−0.0009	−0.010∗	0.6
	(-0.003)	(0.003)	(0.004)	
*Married Low-Educated Mothers with 1 Child*				

Foregone medical visit	0.002	0.005∗	0.010∗∗	0.2
	(0.003)	(0.002)	(0.003)	
Routine medical checkup	0.008∗∗	0.004	−0.002	0.7
	(0.003)	(0.003)	(0.004)	
Dental visit	0.03∗∗∗	0.03∗∗∗	0.03∗∗∗	0.6
	(0.004)	(0.005)	(0.008)	
*Married above High School Educated Mothers with 2 or More Children*				

Foregone medical visit	−0.001	−0.0008	−0.0007	0.1
	(0.001)	(0.0008)	(0.001)	
Routine medical checkup	−0.001	0.002	0.001	0.7
	(0.002)	(0.002)	(0.002)	
Dental visit	0.004	0.002	0.003	0.8
	(0.002)	(0.002)	(0.003)	
*Married above High School Educated Mothers with 1 Child*				

Foregone medical visit	0.004∗∗	0.005∗∗∗	0.005∗∗∗	0.1
	(0.002)	(0.001)	(0.001)	
Routine medical checkup	−0.004	−0.004	−0.002	0.7
	(0.003)	(0.004)	(0.005)	
Dental visit	−0.02∗∗∗	−0.02∗∗∗	−0.02∗∗∗	0.8
	(0.002)	(0.002)	(0.002)	

Note: Each cell represents the effect of a 10 percentage-point increase in refundable state EITCs (relative to federal credits) on the likelihood of an outcome on a 0–1 probability scale; multiplying this coefficient by 100 gives the likelihood change in percentage points. Estimates for forgone care and any routine checkups are based on annual data from BRFSS 1996–2019. Estimate for any dental visits is based on biannual data in even years from 2002 through 2018. Specification (1) includes one EITC variable, average of the refundable percentage of federal credit over 12 months before the interview month depending on which disbursement period the month follows; specification (2) and (3) includes one EITC variable, the refundable percentage of federal credit from the prior tax year (states with no EITC and states with non-refundable ETIC have 0 on this variable as the control group). All models control for state, year, and month of survey fixed effects. The demographic controls include indicators for maternal age, an indicator for mothers of three or more children versus mothers of two children only, race/ethnicity, and education. State level controls include average state minimum wage from the past 12 months, state unemployment rate, state median household family income, maximum state TANF monthly payment for a family of 3, and Medicaid parental eligibility as % FPL. The regression is estimated using weighted least squares using BRFSS sampling weights; standard errors (SE) are clustered at state level and shown in parentheses. ∗*p* < 0.05, ∗∗*p* < 0.01.

There are no clear patterns of effects across these subgroups including for the subgroups with less education and more who would be expected to receive more EITC benefits than the other subgroups with higher education and only one child. For example, for single mothers with high school or less education and one child, there is a small and statistically significant decline in *forgone medical visit* when averaging EITC levels over the past 12 months and including all interview months but not when assigning prior tax year EITC levels and including later interview months (effect in June–November interviews is similar but not statistically significant). And for married mothers with high school or less education and 2 or more children, there is a statistically significant decline in *routine medical checkup* with all three EITC measures and interview months and in *dental visit* when including all interview months and June–November interviews. In contrast, for married mothers with high school or less education and 1 child, there is a statistically significant increase in *dental visit* with all three EITC measures and interview months, increase in *routine medical checkup* when including all months, and increase in *forgone medical visit* with the May–December or June–November interviews. All effect estimates are small and not statistically significant for married mothers with above high school education and 2 or more children. However, there are some estimates indicating worsening outcomes for other subgroups with above high school education expected to receive few EITC benefits, such as an increase in *forgone medical visit* and decline in *dental visit* for married mothers with one child under all three EITC measures and interview months, decline in *dental visit* for single mothers with two or more children when including all interview months or May–December interviews, or decline in *routine medical checkup* for single mothers with one child in all three EITC measures and interview months. Overall, these estimates including the unexpected worsening outcomes in subgroups less likely to be affected by EITC suggest spurious associations and residual confounding in these estimates.

Lastly, Table 5 shows the EITC effect estimates for single childless women with high school or less education, as a placebo check. Under the three EITC measures and interview months, there is a statistically significant increase in *dental visit* and no effects on the other outcomes. This analysis also suggests the possibility of residual confounding when evaluating EITC effects on *dental visit.*

**Table 5:** Effects of a ten-percentage-point increase in refundable state EITC (as % of federal credit) on access to medical and dental care, single low-educated childless women aged 18–44 Years in BRFSS.

Model specifications	(1) Averaging EITC across the past 12 months and including all interview months	(2) Assigning EITC based on prior tax year level and including May–December interviews	(3) Assigning EITC based on prior tax year level and including and including June–November interviews	Outcome Mean
	Effect (SE)	Effect (SE)	Effect (SE)	
Foregone medical visit	0.003	0.002	0.003	0.3
	(0.003)	(0.002)	(0.003)	
Routine medical checkup	0.004	0.003	0.004	0.7
	(0.003)	(0.002)	(0.003)	
Dental visit	0.008∗	0.007∗	0.008∗	0.6
	(0.003)	(0.003)	(0.003)	

Note: Each cell represents the effect of a 10 percentage-point increase in refundable state EITCs (relative to federal credits) on the likelihood of an outcome on a 0–1 probability scale; multiplying this coefficient by 100 gives the likelihood change in percentage points. Estimates for forgone care and any routine checkups are based on annual data from BRFSS 1996–2019. Estimate for any dental visits is based on biannual data in even years from 2002 through 2018. Specification (1) includes one EITC variable, average of the refundable percentage of federal credit over 12 months before the interview month depending on which disbursement period the month follows; specification (2) and (3) includes one EITC variable, the refundable percentage of federal credit from the prior tax year (states with no EITC and states with non-refundable ETIC have 0 on this variable as the control group). All models control for state, year, and month of survey fixed effects. The demographic controls include indicators for maternal age, an indicator for mothers of three or more children versus mothers of two children only, race/ethnicity, and education. State level controls include average state minimum wage from the past 12 months, state unemployment rate, state median household family income, maximum state TANF monthly payment for a family of 3, and Medicaid parental eligibility as % FPL. The regression is estimated using weighted least squares using BRFSS sampling weights; standard errors (SE) are clustered at state level and shown in parentheses. ∗*p* < 0.05, ∗∗*p* < 0.01.

## 4 Discussion

This study is among the first to examine effects of refundable state EITC programs on access to medical and dental care services among single low-income mothers irrespective of pregnancy. We use a national sample from the BRFSS from 1996 to 2019 and leverage extensive variation in refundable state EITC levels over this time while accounting for state time-invariant differences, national trends in outcomes, and several individual-level and state time-varying covariates indicators. Also, we evaluate differences in effects relative to the EITC disbursement period based on interview month. Overall, we find little evidence that an increase in refundable state EITC has effects on access to medical and dental services among the group of mothers mostly likely to benefit from this policy, who are single mothers with high school or less education and two or more children. Even though we find an increase in *dental visit* and decrease in *forgone medical visit* when measuring EITC based on the prior tax year credit level and focusing on later months, the effects are small and inference is sensitive to which interview months are included. Moreover, we find an improvement in *dental visit* in a subgroup that is expected to benefit less from EITC (married mothers with high school or less education and 1 child) and another subgroup with little expected effect if any (childless women with high school or less education), suggesting that the increase in *dental visit* might not be a causal estimate. There are also some unexpected effects suggesting worsening access in other subgroups not expected to be affected much by EITC, further suggesting spurious associations and residual confounding and that effects are not causal. Taken as a whole, the results suggest no discernible effects from EITC on access to medical and dental services among low-income mothers.

The study is also among the first to evaluate and the interactions between refundable EITC and state Medicaid eligibility. We find no evidence of such interactions in this study. Despite the positive sign of all estimated interactions that would suggest reinforcing effects, these estimates were not statistically significant. Examining the interactions between cash transfer policies such as state EITC and Medicaid eligibility for low-income individuals is needed to understand if cash support facilitates the access with having insurance coverage by covering indirect costs of utilizing services such as transportation and childcare., further research considering other income support policies and health care access and utilization measures is important.

The study estimates an intent-to-treat effect of refundable state EITC, i.e. the average effect over the sample irrespective of receiving the credit and amount received. In the main subgroup of mothers with high school or less education and two or more children, the average expected refundable state EITC credit including both recipients and non-recipients is about $500 annually (Qian & Wehby, 2021). This is likely an overall small amount on its own that may result in a small intent-to-treat effect on access to care. As a lumpsum payment, received EITC may be spent differently from employment income and spent within a short duration from disbursement. As access outcomes are measured over the past 12 months, any effects on access may differ not only based on timing but also on when individuals needed or intended to access medical care, which is not observed. Such heterogeneity could be masked in the overall average effect estimates. As such, the findings are not necessarily inconsistent with prior research suggesting positive effects from refundable state EITC on maternal health (Gangopadhyaya et al., 2020; Markowitz et al., 2017; Strully et al., 2010). Such positive effects on maternal health may be due to relieved financial stress. However, other constraints may counter effects of small income increases on access including nonfinancial barriers to care. For example, availability of providers willing to provide care for low-income individuals may be a constraint. Some studies suggest an increase in employment with state EITC (Neumark & Wascher, 2011; Neumark & Williams, 2020; Schmeiser, 2012; Strully et al., 2010). If increased employment reduces time to seek care or reduces Medicaid eligibility for some individuals (especially earlier in the study period when Medicaid eligibility was lower), such effects may further reduce any EITC benefits for access.

Our study contributes to a relatively small body of research on EITC effects on health care access. Prior research has offered mixed findings and largely focused on federal EITC, and studies specific to state EITC and health care access generally focused on pregnant women (Markowitz et al., 2017; Strully et al., 2010). Our study adds evidence on refundable state EITC effects on health care access and utilization among low-income women irrespective of pregnancy. Estimates in models measuring EITC based on the prior tax year credit level and focusing on later months suggest that a $1000 increase in maximum credit translates into a 0.44–1.3 percentage-points reduction in *forgone medical visit*. The magnitude is comparable to previous study examining federal and state EITC combined on access to care (Gangopadhyaya et al., 2020), which suggests a 0.9 percentage-points reduction in *forgone medical visit*. Overall, our findings highlight that improving access in this population requires focusing on supply-side and other demand constraints that impact utilization than interventions that offer small cash payments.

Our study has limitations. Outcomes are self-reported and are subject to reporting errors. However, measurement errors are unlikely to be correlated with state EITC programs and as such are unlikely to bias the effect estimates although may reduce precision (i.e. increase standard errors). Also, we cannot rule out residual confounding possibly due to other state-level economic or policy events contemporaneous to EITC changes. Lastly, we did not have data on the exact EITC amounts that individuals received and the exact timing of disbursement. As noted above, the intent-to-treat effect estimates are policy relevant but may mask individual-level heterogeneity in exposure and effects.

## 5 Conclusion

We find little evidence that an increase in refundable state EITC affects medical and dental care access among low-income mothers including those expected to receive the highest EITC payments considering income and number of children. There is also no evidence that higher EITC levels interact with state Medicaid eligibility in affecting access. Findings suggest that cash support policies providing small lumpsum payments are unlikely to affect access to care. However, these average effect estimates may mask individual-level heterogeneity in treatment effects based on need and timing of health care use relative to EITC amounts and disbursement timing which we leave for future research. Studies that link administrative data on health care utilization such as Medicaid and private insurance claims and on tax filings can capture more precisely individual-level exposure to EITC and health services utilization and the timing of effects.

## CRediT authorship contribution statement

**Haobing Qian:** Writing – review & editing, Writing – original draft, Visualization, Methodology, Analysis, Conceptualization, Project administration, Investigation. **George L. Wehby:** Writing – review & editing, Methodology, Conceptualization, Analysis, Investigation, Supervision.

## Ethical statement

All data is publicly available and de-identified and exempt for human subjects by the University of Iowa IRB.

## Funding

This research did not receive any specific grant from funding agencies in the public, commercial, or not-for-profit sectors.

## Declaration of interests

The authors declare that they have no known competing financial interests or personal relationships that could have appeared to influence the work reported in this paper.

## Data Availability

1996–2019 Behavioral Risk Factor Surveillance System (BRFSS) is publicly available on CDC website: https://www.cdc.gov/brfss/data_documentation/index.htm.
