# Supplementary material for: Effects of refundable state earned income tax credits on access to medical and dental services of low-income mothers
Source: SSM Popul Health. 2025 Mar 19;30:101787. doi: 10.1016/j.ssmph.2025.101787 (PMC11979974; doi:10.1016/j.ssmph.2025.101787)
Supplement: Multimedia component 1 [file mmc1.docx]

APPENDIX

**Supplemental Tables Online Only**

Table S.1 Number of States with Refundable EITC between Tax Years 1995-2018

| **Tax year** | **States with refundable EITC programs** | | |
| --- | --- | --- | --- |
|  | **Number of states** | **Average EITC credits (% of federal credit)** | **Range of state EITC credits (% of federal credit)** |
| 1995 | 4 | 23 | 10-33 |
| 1996 | 4 | 25 | 20-33 |
| 1997 | 5 | 22 | 10-33 |
| 1998 | 6 | 19 | 10-33 |
| 1999 | 7 | 19 | 10-33 |
| 2000 | 9 | 17 | 10-33 |
| 2001 | 9 | 20 | 10-33 |
| 2002 | 9 | 21 | 5-33 |
| 2003 | 11 | 19 | 5-33 |
| 2004 | 11 | 19 | 5-33 |
| 2005 | 12 | 18 | 5-35 |
| 2006 | 13 | 18 | 5-35 |
| 2007 | 15 | 17 | 5-35 |
| 2008 | 18 | 15 | 3.5-40 |
| 2009 | 18 | 16 | 3.5-40 |
| 2010 | 18 | 16 | 3.5-40 |
| 2011 | 19 | 17 | 3.5-40 |
| 2012 | 19 | 16 | 3.5-40 |
| 2013 | 19 | 16 | 3.5-40 |
| 2014 | 19 | 16 | 3.5-40 |
| 2015 | 20 | 16 | 3.5-40 |
| 2016 | 21 | 21 | 3.5-85 |
| 2017 | 22 | 20 | 3.5-85 |
| 2018 | 22 | 22 | 3.5-85 |

Notes: We obtained the annual data of state EITC as percentage of federal credit and refundability status for tax years 2000-2017 from <https://www.taxpolicycenter.org/statistics/state-eitc-percentage-federal-eitc>.

Number of states with refundable EITC programs include Washington, D.C.

Indiana started its state EITC in 1999 but not structured as the federal until 2003 switching to 6% of federal (so it is coded as having a refundable EITC program, but the percentage is coded as missing until 2003). See: <http://www.taxcreditsforworkersandfamilies.org/state-tax-credits/indiana/>

Washington enacted a refundable EITC in tax year 2009 at 5% of federal credit but the program was never implemented due to lack of funds. Therefore, we include Washington as a no EITC state. See: <https://www.ncsl.org/research/labor-and-employment/earned-income-tax-credits-for-working-families.aspx>

Maryland is excluded because its EITC does not follow the federal EITC structure for all years.

South Carolina and Hawaii enacted a nonrefundable EITC at 125% and 20% in tax year 2017, Montana enacted a refundable EITC at 20% in tax year 2017, but the programs were not implemented. Therefore, we include these three states as a no EITC state for the whole study period. See: https://www.taxpolicycenter.org/statistics/state-eitc-percentage-federal-eitc
